# Abnormal primary and permanent dentitions with ectodermal symptoms predict WNT10A deficiency

**DOI:** 10.1186/s12881-016-0349-4

**Published:** 2016-11-24

**Authors:** Birgitta Bergendal, Johanna Norderyd, Xiaolei Zhou, Joakim Klar, Niklas Dahl

**Affiliations:** 1 National Oral Disability Centre for Rare Disorders, The Institute for Postgraduate Dental Education, P.O. Box 1030, SE- 551 11 Jönköping, Sweden; 2 School of Health and Welfare, Jönköping University, Jönköping, Sweden; 3 Department of Immunology, Genetics and Pathology, Uppsala University, Uppsala, Sweden; 4 Science for Life Laboratory, Uppsala University, Uppsala, Sweden

**Keywords:** *WNT10A* mutations, Ectodermal dysplasia, Dental, Primary dentition, Permanent dentition

## Abstract

**Background:**

The WNT10A protein is critical for the development of ectodermal appendages. Variants in the *WNT10A* gene may be associated with a spectrum of ectodermal abnormalities including extensive tooth agenesis.

**Methods:**

In seven patients with severe tooth agenesis we identified anomalies in primary dentition and additional ectodermal symptoms, and assessed *WNT10A* mutations by genetic analysis.

**Results:**

Investigation of primary dentition revealed peg-shaped crowns of primary mandibular incisors and three individuals had agenesis of at least two primary teeth. The permanent dentition was severely affected in all individuals with a mean of 21 missing teeth. Primary teeth were most often present in positions were succedaneous teeth were missing. Furthermore, most existing molars had taurodontism. Light, brittle or coarse hair was reported in all seven individuals, hyperhidrosis of palms and soles in six individuals and nail anomalies in two individuals. The anomalies in primary dentition preceded most of the additional ectodermal symptoms. Genetic analysis revealed that all seven individuals were homozygous or compound heterozygous for *WNT10A* mutations resulting in C107X, E222X and F228I.

**Conclusions:**

We conclude that tooth agenesis and/or peg-shaped crowns of primary mandibular incisors, severe oligodontia of permanent dentition as well as ectodermal symptoms of varying severity may be predictors of bi-allelic *WNT10A* mutations of importance for diagnosis, counselling and follow-up.

## Background

The *WNT10A* (Wingless-type MMTV integration site family, member 10A) gene is a member of the WNT gene family that has been implicated in oncogenesis and in several developmental processes including tooth formation [1, 2]. Bi-allelic *WNT10A* mutations cause a spectrum of symptoms from ectodermal appendages. Patients may present with nail dystrophy, palmoplantar hyperkeratosis, hyperhidrosis as well as hypohidrosis, sparse or brittle hair, eye-lid cysts and keratoconus in different combinations and severity in a continuum of phenotypes [3–6]. In some cases the constellation of features may be defined as a specific clinical entity among the ectodermal dysplasias (EDs), e.g., odonto-onycho-dermal dysplasia (OODD; OMIM 257980) or Schöpf-Schultz-Passarge syndrome (SSPS; OMIM 224750) [3–5, 7]. The most consistent and specific symptom in cases with either bi-allelic or mono-allelic *WNT10A* mutations reported to date is tooth agenesis of the permanent dentition [3, 4, 6, 8–14]. Studies of patients with non-syndromic oligodontia (agenesis of 6–28 teeth) referred for centralized treatments revealed a carrier frequency of up to 56% for *WNT10A* mutations [8]. However, a population-based study of Swedish individuals with isolated oligodontia revealed that the frequency of at least one *WNT10A* mutation in non-related probands was 28% [11]. When adding mutations in the genes *MSX1, PAX9, AXIN2, EDA, EDAR,* and *EDARADD* in the same material the frequency of genetically verified isolated oligodontia reached 38.3%.

Taken together, independent reports from us and others indicate that *WNT10A* mutations are the most frequent specific cause of isolated hypodontia (agenesis of at least one tooth), and oligodontia (agenesis of six or more teeth, third molars excluded) of the permanent dentition known to date. The different frequencies in *WNT10A* associated tooth anomalies in these studies may be related to ethnicity, phenotypic differences and selection criteria of study participants. Noteworthy, mutations in the WNT co-receptor *LRP6* gene were recently identified in rare cases of isolated oligodontia bringing further evidence for the importance of WNT10A in tooth development [15].

Bi-allelic *WNT10A* mutations are usually associated with a larger number of missing teeth when compared to mono-allelic mutations [3, 4, 6, 8–11, 14]. Noteworthy, the penetrance among heterozygotes is incomplete and a considerable proportion of carriers do not express any tooth anomalies at all [12]. In addition to tooth agenesis, *WNT10A* variants may cause abnormal shape of both roots and crowns of permanent teeth [16, 17]. Deformities include taurodontism of molars, misshaped crowns and peg-shaped incisors [10, 14, 16, 17]. Taurodontism is a condition in human molars in which the body of the tooth is enlarged vertically at the expense of the roots, causing the pulp chamber to extend apically below the cementoenamel junction [18, 19]. Thus, isolated hypo- or oligodontia, and tapered frontal permanent teeth give reasons to suspect underlying *WNT10A* mutations. Also, a less disturbed primary dentition with conical frontal teeth was reported by Bohring et al., 2009 [4].

We hypothesized that the combined assessment of primary and permanent dentition as well as search for mild or unnoticed ectodermal symptoms may identify carriers of *WNT10A* mutations. In this study we present seven individuals with severe tooth anomalies in whom symptoms from other ectodermal appendages were identified. We made a detailed characterization of both the primary and permanent dentition together with symptoms from other ectodermal derivatives. Our aim was to evaluate if abnormalities in both primary and permanent dentitions, together with other ectodermal symptoms, may serve as predictors for WNT10A deficiency of diagnostic importance early in life.

## Methods

### Clinical investigations

Seven individuals with oligodontia and other ectodermal symptoms diagnosed at the National Oral Disability Centre for Rare Disorders in Jönköping, Sweden, were retrospectively selected when we identified typical abnormalities in their primary dentition. Two of the patients (Ia and Ib) were siblings and the remaining were probands from five families. Three patients were females and four were males. Clinical data were compiled from patient records including photographs and panoramic radiographs. Evaluation of taurodontism of primary or permanent mandibular molars was performed using the method described by Seow and Lai, 1989 [20], using panoramic radiographs to calculate the quotient of crown-body (CB) and root (R) length. We used the threshold value for taurodontism of 0.9 or more as suggested by Kan et al., 2010 [21]. The measure points for CB and R were fixed by two of the authors separately from the molar segments of the mandible on panoramic radiographs. The accuracy of the measuring points were evaluated simultaneously and adjusted by two specialists in dentomaxillofacial radiology before calculation of the CB:R ratio to establish taurodontism. Ectodermal signs and symptoms were compiled from anamnestic data in patient records and by telephone interviews in order to obtain specific information.

### Genetic analysis

The patients and their parents were sampled and tested for mutations in the *WNT10A* gene. All four exons of *WNT10A*, exon-intron boundaries and 5′ and 3′-UTRs were PCR amplified using primers designed via the Primer3 software (sequences available upon request) and checked with ePCR using the UCSC genome browser. Mutation screening was performed of all coding parts of the *WNT10A* gene by bi-directional Sanger sequencing using Big Dye Terminator v3.1 cycle sequencing kit (Applied Biosystems, Foster City, CA) and separated on an ABI 3730xl DNA analyzer (Applied Biosystems; primers available upon request). Chromatograms were aligned and compared to the reference sequence of the *WNT10A* gene (NM_025216.2).

## Results

### Dental characteristics

Three out of the seven individuals (Ia, II and III) had agenesis of primary teeth (range 2–3) including five maxillary and two mandibular lateral incisors, 5% of all primary teeth in the cohort. In four individuals the mandibular primary incisors were present with peg-shaped crowns. In two individuals (II and V) the primary incisors were severely worn and there was no evidence of the original crown shape, and in one (IV) the primary incisors were lost at time of examination. Thus, hypodontia and/or peg-shaped crowns of primary mandibular incisors were identified in 4 out of 7 patients. Furthermore, existing primary maxillary incisors in patients Ia and III were tapered, though less marked than in the mandible. When comparing genders we observed that the number of missing primary teeth was significantly higher among females (females 2.3 ± 0.6, *n* = 3; males = 0 ± 0, *n* = 4; *p* = 0.020).

**Fig. 1:**
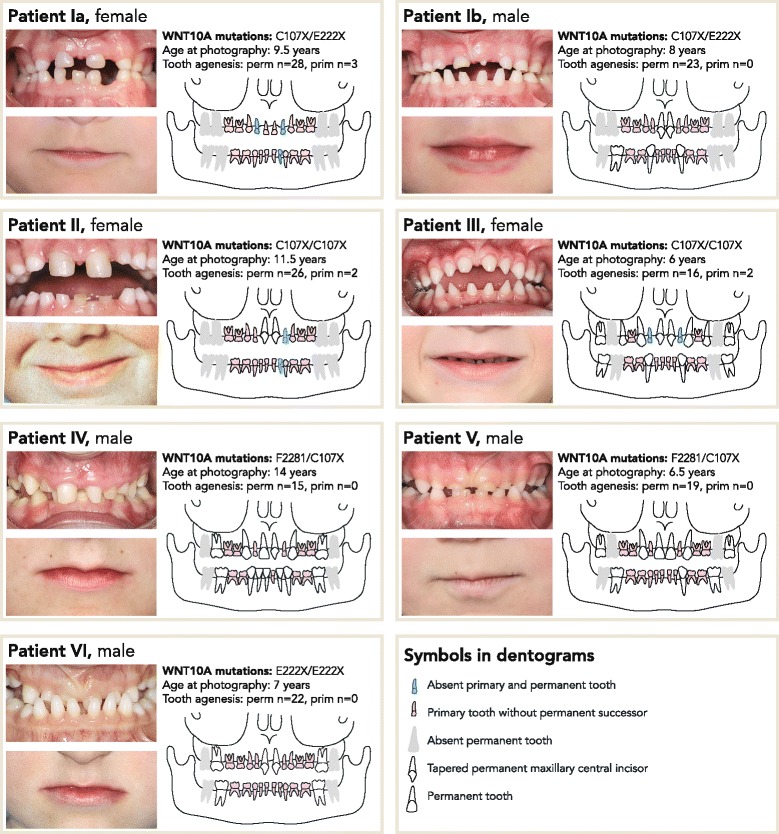
Oral photographs, dentograms and mutations in seven individuals with bi-allelic *WNT10A* mutations. Intraoral photographs show peg-shaped crowns of the primary mandibular incisors (individuals Ia, Ib, III, and IV) and tapered maxillary primary incisors (individuals Ia and III). Extraoral photographs illustrate a characteristic thin upper lip seen in all but individual Ib. Individuals Ia and Ib are siblings

**Fig. 2:**
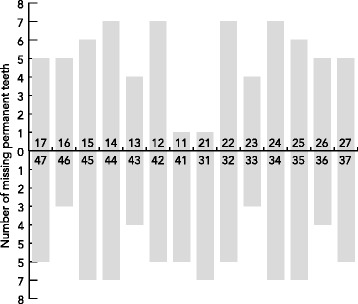
Missing permanent teeth in seven individuals with bi-allelic *WNT10A* mutations (*n* = 149, mean = 21.3, median = 22, range = 15–28). The number of missing teeth was similar in maxilla and mandible

Clinical documentation including intra- and extraoral photographs, dentograms with both primary and permanent dentitions, and *WNT10A* mutations are shown in Fig. 1. Individual Ia had no permanent teeth at all, and individual II had only two permanent maxillary central incisors. Examination of the permanent dentition revealed extensive oligodontia with agenesis of 76% of permanent teeth (Fig. 2.). The mean number of missing permanent teeth in all seven individuals was 21.3 (range 15–28) with a distribution of 47% in the maxilla and 53% in the mandible. In the six individuals with some permanent teeth, five had severely tapered maxillary central incisors, nine out of 12 teeth (75%). All seven individuals were missing maxillary lateral incisors, first premolars of both jaws, and mandibular second molars and left central incisors. The most stable teeth, present in all but in individual Ia, were the maxillary central incisors, followed by the canines in both jaws. There was no difference in number of missing permanent teeth when comparing males to females.

Taurodontism was investigated in mandibular molars using panoramic radiographs. In total, 11 primary second molars were possible to examine in six individuals; in individual IV the primary molars were partly resorbed. Nine primary molars, 82%, had a score of 0.9 or more. Consequently, six individuals had taurodontism in one or both primary molars. Taurodontism of permanent dentition was confirmed in individual IV where the first molars were fully erupted. He was the only individual where evident taurodontism could be determined by cursory examination. Clinical data are summarized in Table 1.

**Table 1:** Clinical data in seven individuals with bi-allelic *WNT10A* mutations

Patients	Ia	Ib	II	III	IV	V	VI	Total
Age at radiographic examination	8.5	7.5	8.0	6.0	16.5	6.5	7.0	
Tooth agenesis in primary dentition	3	0	2	2	0	0	0	7
Tooth agenesis in permanent dentition	28	23	26	16	15	19	22	149
Tapered maxillary central incisors	-	2	2	2	1	0	2	9
Molars available for analysis of taurodontism^a^	75	75	75	-	36	75	75	
	85	85	85	85	46	85	85	13
Molars with taurodontism^a^	1	2	2	1	2	1	2	11
Hyperhidrosis of palms and soles	+	+	-	+	-	+	+	5
Coarse hair structure and/or light/scarce/brittle hair	+	+	+	+	+	+	+	7
Nail abnormalities	-	-	-	-	+	-	+	2
Dry skin	+	+	+	+	-	+	+	6
No. of affected ectodermal appendages	3	3	2	3	2	3	4	mean 2.9

^a^All were primary molars except for individual IV who had resorbed primary second molars and erupted permanent first molars

### Additional ectodermal abnormalities

Ectodermal symptoms other than tooth agenesis were in some cases unnoticed, neglected or considered normal variants by the patient/parents. Upon interview, six individuals reported to have dry skin, and the number of affected ectodermal appendages in each patient varied from 2 to 4 (mean 2.9, median 3) (Table 1). Hyperhidrosis of palms/soles was reported from five patients, with most severe symptoms in individual Ia who also showed palmo-plantar hyperkeratosis. All seven patients reported on hair abnormalities including coarse, brittle and scarce hair. Slow-growing hair was reported in individuals Ia and II with agenesis of all or almost all permanent teeth, respectively. Abnormal nail phenotype was reported in two individuals: Individual IV who had absent nails at birth and individual VI who had mild hyponychia and brittle nails. None of the patients could be classified as a specific ED entity [22].

Unexpectedly, 6 patients shared a facial feature of a thin upper lip seen in all but individual Ib (Fig. 1.). Furthermore, individual IV had a dished-in facial profile as reported previously in association with WNT10A-associated tooth agenesis [14]. No patient reported any systemic conditions.

### Genotyping

Genetic analysis of exons and their flanking sequences of *WNT10A* was performed as described previously [11], and the results revealed bi-allelic mutations in all seven individuals. All parents were found to be heterozygous. Altogether, three different mutated alleles were identified resulting in p.C107X, p.F228I and p.E222X (Fig. 1). These three WNT10A variants have been reported earlier [4, 9].

## Discussion

We investigated seven individuals with severe oligodontia for their primary dentition, presence of ectodermal symptoms and *WNT10A* mutations. The selection of patients was based on severe oligodontia of permanent dentition and suspected but yet unspecified ED. Following assessment of primary dentition and presence of other ectodermal symptoms, the patients were analyzed for *WNT10A* gene variants. Bi-allelic mutations were confirmed in all seven individuals. In total, three different *WNT10A* alleles were detected that predict the previously described variants C107X, E222X and F228I. Clinical investigations at age 6–14 years revealed that the most distinctive initial sign in all examined individuals was the presence of numerous primary teeth in areas without succedaneous teeth. In total, seven primary incisors were missing from agenesis in three out of seven individuals, 5% of all primary teeth, compared to 76% of permanent teeth (mean 21.3). Notably, all three females but none of the males had primary tooth agenesis. This observed gender difference for absent primary teeth is significant (*p* = 0.02) but needs to be confirmed in larger and randomized cohorts. The most stable teeth were the maxillary central incisors, which were missing in only one individual. Severe dental abnormalities were found in individual Ia, who was missing three primary teeth and all permanent teeth, as well as individual II, missing two primary teeth and 26 permanent teeth, all but the maxillary central incisors.

Notably, all well-preserved primary incisors examined were peg teeth. Similarly, the permanent maxillary central incisors were severely tapered in 75% that were present. Taurodontism of primary or permanent molars was found in all of the examined individuals using biometric analysis from panoramic radiographs. This method is superior and more sensitive than cursory examination, especially in cases of milder forms of taurodontism [23]. The most evident signs were found in the single individual in whom permanent molars were assessed. In mandibular molars from either the primary or permanent dentitions present in each individual 84.6% were taurodontic. However, taurodontism is frequently found in individuals with hypodontia [21], with or without ED.

Besides tooth agenesis and misshaped teeth, all patients presented with an overlapping spectrum of mostly mild or previously unnoticed ectodermal symptoms including hyperhidrosis of palms and soles, aberrant hair texture, dry skin and nail dystrophy. None of the patients presented with the typical phenotype of *EDA*- or *EDAR*-induced hypohidrotic ED [24, 25] and the ectodermal signs and symptoms were incongruous with any defined ED-entity. The spectrum of ectodermal symptoms among our seven patients is consistent with that previously reported in patients with bi-allelic *WNT10A* mutations [3–6, 8, 10], and even in some patients with mono-allelic mutations [4, 6, 8, 11, 12]. Notably, with the exception of tooth agenesis, the signs and symptoms from ectodermal appendages in a majority of individuals in our cohort were subtle and in several cases even not experienced as abnormal by the study persons or their parents. Furthermore, we confirmed that carriers for bi-allelic *WNT10A* mutations have more primary teeth in positions where permanent teeth are missing [4]. Thus, in children with severe tooth agenesis it is important to consider mild or neglected symptoms from other ectodermal appendages. The molecular basis for the broad range of clinical expression due to WNT10A deficiency, from completely asymptomatic carriers to severely disabling ED entities, is yet unclear [10]. Interestingly, a thin upper lip was observed in all but one individual in our study. We observed a similar feature in photographs in three out of five patients from the study by Bohring et al., 2009 [4] Supplemental data]. This sign is distinct from the typical facial phenotype of *EDA*- and *EDAR*-induced ED and further studies are now needed to confirm this observation in carriers of *WNT10A* mutations.

Besides the critical role in the development of ectodermal lineages, increased expression of *WNT10A* has previously been implicated in a variety of cancers by up-regulation of the β-catenin pathway [1, 26]. In contrast, the structural *WNT10A* mutations known to be associated with abnormal development of ectodermal tissues are predicted to disrupt or reduce WNT mediated signaling [27]. Taken together, this is consistent with the fact that patients with structural *WNT10A* mutations are not reported to have increased risk for cancer.

Our study shows that bi-allelic *WNT10A* mutations are associated with a full or nearly full set of primary teeth with typical peg-shaped crowns of the mandibular incisors, persisting primary teeth in areas with missing permanent successors, and extensive tooth agenesis (oligodontia) in the permanent dentition, often with severely tapered maxillary central incisors. This is consistent with and adds to the dental features described by Bohring et al., 2009 [4], Supplemental data]. Furthermore, in combination with mild abnormalities in other ectodermal appendages, this typical dental pattern strongly suggests a *WNT10A*-associated ED that differs from what is seen in *EDA-* and *EDAR*-induced hypohidrotic ED.

## Conclusions

We conclude that overt abnormalities in both primary and permanent dentitions together with other ectodermal symptoms can serve as predictors for WNT10A deficiency. Thus, individuals with bi-allelic *WNT10A* mutations may be recognized early in life by combining information of both dentitions as well as other ectodermal features of importance for diagnosis, prognosis and counselling.

## Abbreviations

CB:R: Crown-body:root length
ED: Ectodermal dysplasia
PCR: Polymerase chain reaction
UCSC: University of California Santa Cruz
UTR: Untranslated region
